# Modifying the Meaning of the Upper Anchor Predictably Shapes Ratings of Perceived Effort: A Randomized Crossover Trial

**DOI:** 10.1186/s40798-026-01040-x

**Published:** 2026-06-06

**Authors:** Dennis Okhamafe, Yedidya Silverman, Andrew D. Vigotsky, Israel Halperin

**Affiliations:** 1 Department of Health Promotion, School of Public Health, Gray Faculty of Medicine and Health Sciences, Tel-Aviv University, Tel-Aviv, Israel; 2 Sylvan Adams Sports Science Institute, Tel Aviv University, Tel-Aviv, Israel; 3 Neuroscience Center, University of North Carolina at Chapel Hill, Chapel Hill, NC USA; 4 Department of Cell Biology & Physiology, University of North Carolina at Chapel Hill, Chapel Hill, NC USA; 5 Department of Pharmacology, University of North Carolina at Chapel Hill, Chapel Hill, NC USA

**Keywords:** Effort, RPE, Anchoring procedures, Isometric contraction

## Abstract

**Background:**

Ratings of perceived effort (RPE) are used to prescribe and monitor training. Yet, the influence of how the upper anchor (“10”) is defined remains underexplored, despite holding theoretical and practical value. Here, we examined whether modifying the upper anchor predictably modifies RPEs during isometric exercises.

**Methods:**

We conducted a within-subject, randomized crossover study in 26 resistance-trained adults. Participants performed isometric contractions at 20–100% of maximal voluntary contraction (MVC) in three exercises: unilateral plantar flexion, unilateral knee extension, and the isometric mid-thigh pull. After each contraction, participants reported RPE (0–10) relative to one session-specific anchor: same-task (maximal effort in the task performed), different-task (maximal effort in the isometric mid-thigh pull), or self-selected (the most effortful task ever experienced or imagined). We estimated expected RPEs and slopes relating RPE to %MVC for each exercise–anchor combination. Preregistered comparisons assessed anchor effects, muscle-mass dose–response, and approximate scale invariance across anchors.

**Results:**

Anchor type substantially influenced RPE. Same-task anchors consistently produced the highest RPEs, self-selected the lowest, and different-task anchors intermediate values, with effects most pronounced at 80–100% MVC. The ratings increased with %MVC across all conditions. Under the self-selected anchors, exercises engaging greater muscle mass tended to yield higher RPEs. The relative differences between exercises were largely preserved across anchors, indicating that anchor choice alters RPE while maintaining approximate scale invariance.

**Conclusions:**

Upper anchors predictably shape RPE across tasks and intensities. Researchers and applied practitioners using RPE in resistance training should explicitly define, justify, and consistently report anchoring procedures, and align anchor choice with the intended purpose to support valid interpretation and comparisons.

**Supplementary Information:**

The online version contains supplementary material available at 10.1186/s40798-026-01040-x.

## Key Points

- Changing the upper anchor on RPE scales can reframe how people rate their efforts.
- We compared three anchor types across exercises differing in muscle mass involvement.
- Anchor type substantially influenced RPE in a consistent and predictable manner.
- Clear anchoring procedures are essential for accurate, replicable, and interpretable findings.

**Supplementary Information:**

The online version contains supplementary material available at 10.1186/s40798-026-01040-x.

## Background

Rating of Perceived Effort (RPE) scales are frequently utilized by exercise and sports scientists, coaches, and trainees to prescribe and monitor exercise intensity [1–5]. RPE scores show moderate to strong associations with concurrently measured physiological outcomes [6–8], such as heart rate and blood lactate [6, 8], as well as with performance-related outcomes [4, 5, 9–12], such as movement velocity [9, 10] and lifted load [10]. These relationships are maintained across diverse populations [2, 4, 13, 14] and exercise modalities, including resistance training [5, 10], endurance exercise [13, 15], and team sports [3]. Over the years, various definitions of effort and perceived effort have emerged [16–21], along with different instructions and scales [16, 22–27]. These developments have led to inconsistent definitions and thus implementations of RPE, both in research and in practice [1, 17, 20, 21]. Several reviews have discussed ways to reduce these inconsistencies [1, 17]. Yet, despite these efforts, one critical aspect has received comparatively little attention: the anchoring procedures used to establish the upper end of the different RPE scales (*i.e.*, the meaning of “10” or “20” on a 0–10 or 6–20 scale) [1, 17].

A potential reason for the limited research on anchoring procedures may stem from how they have been conceptualized and studied, specifically by contrasting exercise-based and memory-based approaches [28–32]. In the exercise-based method, participants first perform the same task they will later complete during the experiment at maximal effort and use that experience as the anchor. In the memory-based method, participants are asked to recall or imagine performing the experimental task at maximal effort and to use that recollection as the anchor. Studies comparing these approaches have reported statistically and practically negligible differences in RPEs, typically less than or equal to one RPE unit on a 6–20 scale [28, 30–32], which likely contribute to the diminished interest in this area of research. However, the similar ratings may be a result of the anchor task being identical in both cases (*e.g.,* lifting a heavy object versus recalling lifting a heavy object). In contrast, many studies use self-selected anchors [33–37], in which participants are instructed to imagine or recall the most intense effort they have ever experienced. Employing self-selected anchors implies that participants will often rely on anchor tasks that differ from the task performed during the experiment, contrary to how anchoring has typically been examined. This distinction is crucial, as RPEs may vary depending on the anchor used.

To address this gap, Malleron et al. [38] proposed distinguishing between imposed and self-selected anchors and examined the effect of this distinction in a within-subject crossover study. Under an imposed condition, participants were instructed to use a specific experimenter-defined task performed at maximal effort as their anchor: maximal voluntary isometric contraction (MVC) using a handgrip or during an isometric squat. In contrast, under a self-selected condition, participants were required to choose the most effortful task they had ever experienced or could imagine. We note that participants reported using a range of anchors, including childbirth and loaded military marches. The effects of these anchoring methods were compared under both fatiguing and non-fatiguing conditions. Two key findings were identified: (1) imposed anchors consistently yielded higher RPE scores, often reaching maximal values regardless of task or fatigue; and (2) self-selected anchors produced higher RPE values during the isometric squat than during the handgrip, particularly as repetitions progressed and fatigue accumulated. Together, these findings highlight that anchoring procedures can saliently affect RPE scores and suggest that anchoring effects may be predictable and interpretable.

While Malleron et al. advanced the conceptualization of anchoring procedures, their work raises key questions. Methodologically, they examined a narrow set of conditions: two tasks performed at repeated, maximal efforts. It remains unclear whether their findings extend to more typical, submaximal exercise contexts. Conceptually, Malleron et al. observed that anchors representing tasks requiring greater effort (*i.e.*, relatively higher energy expenditure; hereafter referred to as *harder tasks*) yielded lower RPE scores than anchors representing tasks requiring less effort (*i.e.*, relatively lower energy expenditure; hereafter referred to as *easier tasks*). This pattern suggests that individuals “reframe” their ratings relative to the perceived effort level implied by the anchor. In other words, a submaximal isometric contraction would elicit a lower RPE when the anchor represents a harder task, such as a maximal isometric squat (*i.e.*, higher energy expenditure), compared with when the anchor represents an easier task, such as a maximal handgrip contraction (*i.e.*, lower energy expenditure). A simple hypothesis is that this “reframing” acts as a linear rescaling of RPE*: the anchor rescales the ratings but not the relative difference (ratio) between exercises.* If this hypothesis holds, then the relative differences between exercises should remain constant across anchors. For example, if exercise A elicits twice the RPE of exercise B at a given relative effort and anchor, then exercise A should also have twice the RPE of exercise B when the anchor changes. Testing this hypothesis requires a more sophisticated study design than that used by Malleron et al.

The current study was designed to address these gaps. First, we assessed RPE across a wide range of intensities (20–100% MVC), extending beyond the maximal efforts previously examined. Second, we evaluated RPE across three tasks that differed in the degree of muscle mass involvement, allowing us to examine the dose-dependent relationship between muscle mass and RPE. Third, in addition to the two anchors used by Malleron et al. (imposed same-task: maximal effort in the task performed; and self-selected: the most effortful task ever experienced or imagined), we introduced a third: an imposed different-task: maximal effort in a task that is not currently being performed. Having multiple anchors enabled us to test whether RPE scores vary systematically with the supplied anchor.

Our hypothesis predicts that (1) RPE would increase with relative force level; (2) different anchors would systematically alter RPE values, with self-selected anchors yielding the lowest scores, imposed same-task the highest, and imposed different-task intermediate scores; (3) exercises that recruit more muscle would produce higher RPE ratings, reflecting a dose-dependent effect in both the self-selected and imposed different-task anchors; and (4) relative differences in RPE across tasks would remain similar when using self-selected and imposed different-task anchors, reflecting scale invariance.

## Methods

### Participants

We recruited a sample of 26 resistance-trained individuals (13 M, 13 F) via posts on various social media platforms. The inclusion criteria included being between 18 and 40 years old and having at least one year of resistance training (RT) experience. Participants were required to have previous experience with performing the plantar flexion, knee extension, and deadlift exercises. Demographic information is presented in Table 1. Informed consent was obtained prior to the first session.

**Table 1:** Participants' characteristics

Characteristic	Male (*N* = 13)	Female (*N* = 13)
Age [yrs]	29.4 ± 4.7	29.9 ± 4.8
Height [cm]	176 ± 8.9	163.9 ± 6.3
Weight [kg]	76.3 ± 12.2	68.5 ± 15.4
Lean body mass [kg]	63.3 ± 8.0	47.7 ± 5.8
Training experience [yrs]	6.4 ± 3.7	6.8 ± 4.9
PF peak torque (N·m)	158.3 ± 32.6	122.5 ± 37.4
Relative PF peak torque (N·m/kg)	2.07 ± 0.4	1.79 ± 0.6
KE peak torque (N·m)	257 ± 51.8	194.7 ± 41
Relative KE peak torque (N·m/kg)	3.37 ± 0.7	2.84 ± 0.6
IMTP peak force (N)	2017.3 ± 529.4	1482.5 ± 359.2
Relative IMTP peak force (N/kg)	26.44 ± 6.9	21.63 ± 5.2

All values are presented as mean ± SD. Isometric plantar flexion (PF). Isometric knee extension (KE). Isometric mid-thigh pull (IMTP)

### Sample Size

We aimed to recruit 18 or 30 participants, depending on the initial results (see pre-registration document: https://aspredicted.org/gm6g-rtp9.pdf). These targets were based on three considerations. First, the ceiling (30) reflected our recruitment capacity and available resources [39]. Second, they were informed by variance estimates from our previous work that examined similar interventions in a comparable population [38]. Third, these candidate sample sizes allowed us to assign participants evenly across conditions, ensuring a balanced number of participants per anchor order group. We note, however, that we concluded the study with 26 participants because recruitment became increasingly difficult, meaning that our final sample size was ultimately determined by practical constraints.

### Procedures

**Fig. 1:**
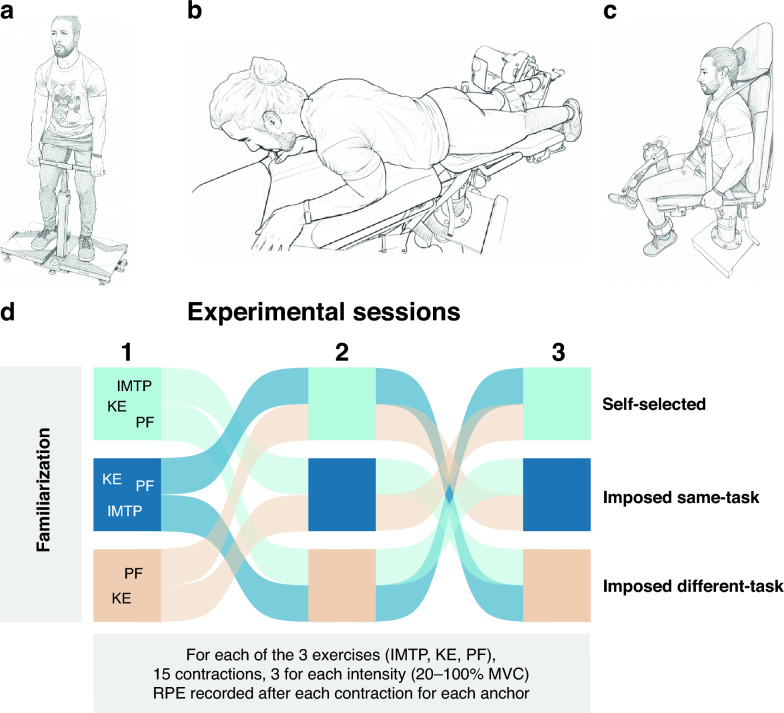
Experimental setup and schematic overview of the randomized crossover design. **a** Isometric plantar flexion (PF). **b** Isometric knee extension (KE). **c** Isometric mid-thigh pull (IMTP). **d** Visual summary of the study design. Each colored square represents one of the three RPE anchoring conditions: self-selected, imposed same-task, and imposed different-task. Colored paths illustrate the six possible session orders to which participants were block-randomized. For each exercise, participants performed 15 five-second contractions (three per intensity: 20%, 40%, 60%, 80%, and 100% of MVC), with RPE recorded after each contraction. The order of exercises and contraction intensities was randomized across participants

We employed a within-subject, randomized crossover design. Each participant attended four laboratory sessions: one familiarization and three experimental sessions, spaced 2–14 days apart. During each experimental session, participants performed isometric contractions at varying intensities across three exercises: isometric plantar flexion (PF), isometric knee extension (KE) (both using an HUMAC NORM isokinetic dynamometer, CSMi, Stoughton, MA, USA), and isometric mid-thigh pull (IMTP) (K-Delta force plate, Kinvent, Orsay, France). After each repetition, participants reported their RPE. To standardize and increase measurement accuracy, participants lay prone for PF and sat for KE, with the dominant leg (active limb) securely attached to the dynamometer. In the IMTP, the handle height was adjusted to mid-thigh level. These settings were recorded during the familiarization session and replicated throughout the experimental sessions (Fig. 1a–c).

Before each contraction, slack was removed, and participants applied force steadily against the plate (PF), pad (KE), or by pulling the handle with their hands while pushing their feet into the ground (IMTP), avoiding explosive movements (for more information, see Additional file 1). Each experimental session corresponded to one of three RPE anchor conditions: same-task, different-task, or self-selected (described below). The assignment of RPE anchor conditions was block-randomized and counterbalanced, such that each participant was assigned to one of six possible permutations of anchor conditions and their order (*e.g.,* same–different–self-selected, self-selected–same–different, etc*.*). Each permutation was tested on four or five participants. To minimize potential order effects, the sequence of the three exercises (PF, KE, and IMTP) and the order of repetition intensities were further randomized for each participant within every experimental session using an online randomization tool (https://www.random.org/lists). The only exception was the different-task anchor condition, in which the IMTP was consistently performed first (as described below), followed by a randomized order of the PF and KE exercises (Fig. 1d).

All sessions followed the same sequence: a general warm-up, an exercise-specific warm-up, a maximal voluntary contraction (MVC) test for the session's designated exercises, and the experimental protocol. The general warm-up consisted of three continuous rounds of knee lifts, heel flicks, jumping jacks, and bodyweight squats, each performed for 10 s, totaling two minutes. Following this, participants completed a 60-s specific warm-up, during which they practiced applying, maintaining, and monitoring force production. As part of this warm-up, they performed 5-s isometric contractions at 20%, 40%, and 60% of either normative force values [40, 41] or force values consistent with prior research in our laboratory [42]. Next, participants performed three 5-s MVCs with one minute of rest between trials. We decided to withhold both verbal and visual feedback during the MVCs to minimize bias. First, verbal encouragement is difficult to standardize across participants and sessions, and even small differences in tone, timing, or intensity may influence force output [43]. Second, visual feedback may have allowed participants to calibrate the force they produced relative to their MVC and potentially align their ratings with the displayed percentage rather than their perceived effort. For example, inferring that a contraction near 90% of MVC could prompt a response such as “9”, independent of the perceptual state. The first MVC served as an additional warm-up, while the highest peak force from the remaining two trials was used to determine the target force levels for that session’s experimental tasks. Participants then completed the experimental protocol corresponding to the assigned RPE anchor condition for that session.

Participants performed the exercises in a randomized order, with a 10-min rest between exercises. For each exercise, participants completed 15 five-second isometric contractions in a randomized order: three repetitions at each of the following intensities: 20%, 40%, 60%, 80%, and 100% of that session’s MVC. In the PF and KE tasks, both the target absolute force (with a ± 5% tolerance range) and the participant’s ongoing absolute force output were displayed as horizontal lines on a screen. In the IMTP, the absolute force output was displayed on a circular scale with numerical values. Before each contraction, participants were verbally informed of the target force required. This setup allowed real-time visual feedback and monitoring (for more detailed information, see Additional file 1). Participants were not informed of their MVC values to minimize rating bias. Accordingly, when the *y*-axis displays absolute force for each contraction, it would be difficult to infer the corresponding percentage of MVC, thereby preventing them from aligning their RPEs with expected values (*e.g.,* associating 60% of MVC with an RPE of 6). Participants rested for 20 s between repetitions and reported their RPE immediately after each repetition using the session's assigned anchor. Rating was accomplished using a 0–10 scale where 0/10 represented the complete absence of effort, and 10/10 signified maximal effort (see Additional file 1).

### Familiarization (Session 1)

At the start of the familiarization session, participants’ weight, height, and body composition were measured (SECA® mBCA 515 and 274, Hamburg, Germany). They then received a 25-min presentation explaining the concepts of effort and perception of effort, the RPE scale, and the three anchor conditions (for further elaboration, see Additional file 1). The experimental procedures were then reviewed verbally in detail to ensure full understanding. Finally, participants completed a general warm-up, followed by the research exercises in a fixed order: IMTP, PF, and KE. For each exercise, they performed a specific warm-up, three MVCs, and a modified version of the experimental protocol. For each exercise, participants practiced two of the three RPE anchoring conditions. For example, during the PF, participants rated their RPE using the same-task and different-task anchors; during the KE, they used the different-task and self-selected anchors. Anchor assignments were randomized and counterbalanced to ensure that each anchor was used twice across the familiarization session.

Although all three anchors were practiced in the PF and KE exercises, the different-task anchor was not applied during the IMTP. This omission was intentional, as the IMTP served as the reference for the different-task anchor across all exercises, making its use in the IMTP equivalent to the same-task anchor and therefore redundant. During each anchoring condition, participants performed three 5-s isometric contractions per exercise at intensities corresponding to 40%, 60%, and 80% of their previously measured MVC, with 20 s of rest between repetitions. After each contraction, they reported their RPE according to the assigned anchor. Following a one-minute rest, the same procedure was repeated using the second anchor for that exercise. A 10-min rest period was provided between exercises.

### Experimental Sessions (Sessions 2–4)

The key distinction between experimental sessions lay in how the upper anchor on the RPE scale was defined. That is, the meaning assigned to a rating of 10/10:

- In the same-task condition, 10/10 reflected the maximal perceived effort of the exercise being performed.
- In the different-task condition, 10/10 referred to maximal perceived effort during a maximal IMTP, regardless of the exercise performed during the session.
- In the self-selected condition, 10/10 represented the most effortful task the participant had ever experienced or could imagine performing.

At the beginning of each session, participants received a brief explanation and reminder about how to interpret and apply RPE ratings according to the assigned anchoring condition. They then completed the full experimental protocol corresponding to that session’s anchor. In the same-task and self-selected sessions, participants performed all three exercises. In contrast, during the different-task session, participants completed only the MVC test for the IMTP, which served as the reference anchor for the task. They did not perform the full contraction protocol for the IMTP itself, as it was the anchor task, making its inclusion in the exercise sequence redundant. Aside from this adjustment, all other procedures were identical across sessions.

## Measures

### Primary Outcomes

RPE**:** Reported immediately following each repetition (15 ratings per exercise). Measured using a 0–10 numerical rating scale with 10/10 corresponding to the session-specific anchoring condition (see Additional file 1).

### Secondary Outcomes

Qualitative data: After completing the self-selected anchor condition, participants verbally answered three open questions (all responses were recorded and later transcribed for analysis): (1) "Which task did you recall or imagine to represent maximal effort?" (2) "Was the task previously experienced or imagined?" (3) "In familiarization, did you use the same anchor? If not, which task was it?" (see Additional file 1).

After completing the final session, participants answered the following three closed-ended questions, each rated on a 7-point Likert scale (0 = not at all; 6 = very much), assessing the extent to which they could distinguish between (1) same-task and different-task anchors, (2) same-task and self-selected anchors, and (3) different-task and self-selected anchors. Participants also verbally answered the following open-ended question: "To what extent do you think using a certain anchor (same-task, different-task, or self-selected) affected your perception of effort in real-time when performing the exercises?" (see Additional file 1).

### Statistical Analysis

We took multiple analytic approaches to assess how anchoring affects RPE. For all analyses, we averaged each individual’s RPEs across each exercise–anchor–intensity triple. First, we visually inspected the averaged data by conditioning on participant, exercise, anchor, and intensity. This visualization provided a sense of which aspects of the data may be important to consider in our models.

Second, we aimed to precisely quantify (model) the RPEs for each condition without imposing strong parametric constraints, *e.g.*, a linear effect of %MVC. To this end, we fit a Bayesian multilevel model with a factor analytic random effects structure [44]. This model parsimoniously reduces the 40 × 40 random effects variance–covariance matrix (820 parameters) into a factor model with five factors, corresponding to the intercept, exercise, anchor, intensity (MVC), and an unconstrained factor [44]. Error variance was permitted to vary freely across all exercise–anchor–intensity triples. In this work, we will not examine the factor analytic structure in detail; instead, we will focus on the so-called fixed effects—*i.e.*, the expected RPE for each exercise, anchor, and intensity, adjusted for the inter-individual variation captured by the factor analytic random effects structure. Although this model faithfully represents the data, it yields 40 fixed effects estimates, making it difficult to succinctly compare relative RPE changes across conditions in a way that is consistent with our preregistered hypothesis.

Our second model was used to parsimoniously reduce our findings to a single parameter for each exercise–anchor pair: a slope, α, that relates RPE to %MVC $\mathrm{RPE} = \alpha \, (\mathrm{\%MVC})$. The slope parameters were then used to address our four predictions: (1) RPE increases with force level (all $\alpha > 0$); (2) anchors systematically and predictably alter RPE, with anchors representing harder tasks leading to lower RPE scores $(\alpha\, [\mathrm{self-selected}] < \alpha\, [\mathrm{different-task}] < \alpha\, [\mathrm{same-task}])$. Note that in this context, *easier tasks* refer to those engaging less muscle mass than *harder tasks* when performed at comparable relative intensities, as they require less energy to execute; (3) for a given anchor, RPE scores are greater in harder tasks than in easier tasks $(\alpha\, [\text{isometric mid-thigh pull}] > \alpha\, [\text{knee extension}] > \alpha\, [\text{plantar flexion}]$α[isometric mid-thigh pull] > α[knee extension] > α[plantar flexion]); and (4) the ratios between the RPE’s from different tasks would remain similar across anchors ($\frac{\alpha [\text{plantar flexion, self-selected}]}{\alpha [\text{knee extension, self-selected}]}\approx \frac{\alpha [\text{plantar flexion, different-task}]}{\alpha [\text{knee extension, different-task}]}$). To estimate these *α*'s, we fit a Bayesian multilevel model. We parameterized *α* with a fixed effect for each exercise–anchor pair and with random effects using a four factor model: an intercept factor, an exercise factor, an anchor factor, and an unconstrained factor. Error variance was permitted to vary freely across all exercise–anchor–intensity triples. We used the posterior distribution of each fixed effect to facilitate comparisons. Data preprocessing and postprocessing were performed in R (R Core Team, Vienna, Austria, version 4.6.0, 2026), and the models were fit using Stan (cmdstanr 0.9.0).

## Results

### Description of Ratings

**Fig. 2:**
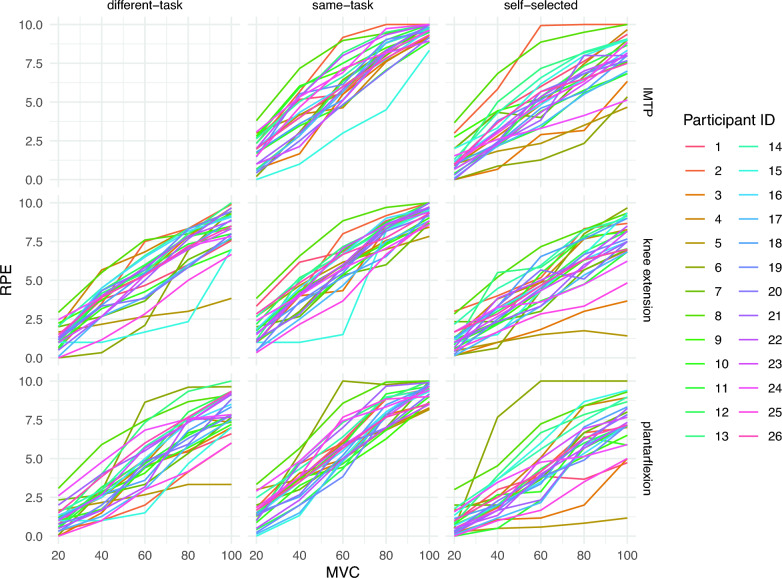
Depictions of individual-level RPE–MVC relationships. The data from each subject were averaged over each exercise–anchor–intensity triple and plotted

Figure 2 depicts the ratings for each participant across all conditions. Even without statistical modeling, some clear trends are apparent. For example, there are strong, monotonic relationships between MVC and RPE, and RPEs are lowest with the self-selected anchor. These plots, which include individual-level data, should provide the reader with a clear picture of the trends that our models aim to capture.

### Modeling of Ratings

Model 1: Our first model treated MVC categorically to estimate the expected RPE for each exercise–anchor pair at each MVC, without assumptions concerning the shape of the RPE–MVC relationship. The posterior chains demonstrated good mixing and model fit the data well (posterior mean ± SD: inverse variance-weighted root-mean square error [wRMSE] = 0.60 ± 0.05). Since we will not heavily rely on this model for our inferences, a visual representation of its results is provided in Additional file 1.

**Fig. 3:**
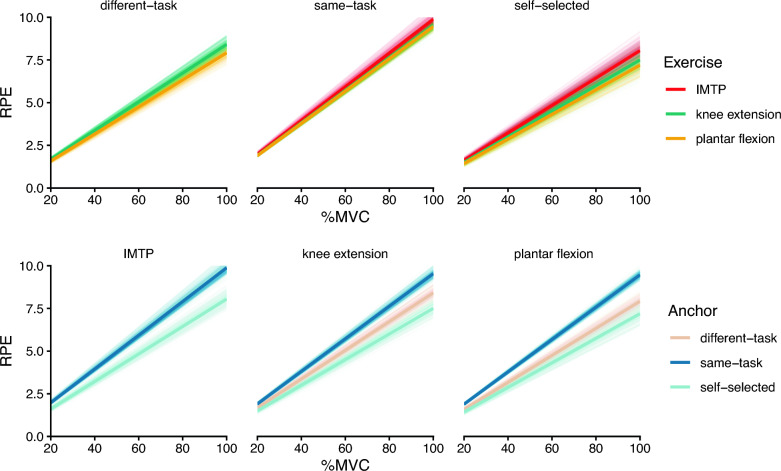
Posterior distributions of the expected linear relationship between MVC and RPE for each exercise–anchor pair after accounting for across-participant differences. The thick line represents the posterior mean of the fixed effect, and the translucent lines represent posterior draws reflecting the uncertainty around the posterior mean. (Top row) Each facet that conditions on anchor emphasizes the effect of exercise (color); (bottom row) conditioning on exercise emphasizes the effect of anchor (color). Generally, the effects of exercise appeared modest (different-task and self-selected) to negligible (same-task), while the anchor effects were more dramatic. These effects can be more readily assessed by investigating the slopes themselves

Model 2: In all cases, exercises’ RPEs increased monotonically, and almost linearly, with respect to %MVC (Fig. 2). Thus, we modeled RPE as a linear scaling of %MVC, *i.e.*, $\mathrm{RPE} = \alpha \, \left(\mathrm{\%MVC}\right)$RPE = α (%MVC) (Fig. 3). The Markov chains used to generate the posterior samples demonstrated good mixing and model fit the data well (posterior mean ± SD: inverse variance-weighted root-mean square error [wRMSE] = 0.79 ± 0.09), indicating that the estimates should provide faithful inferences.

**Fig. 4:**
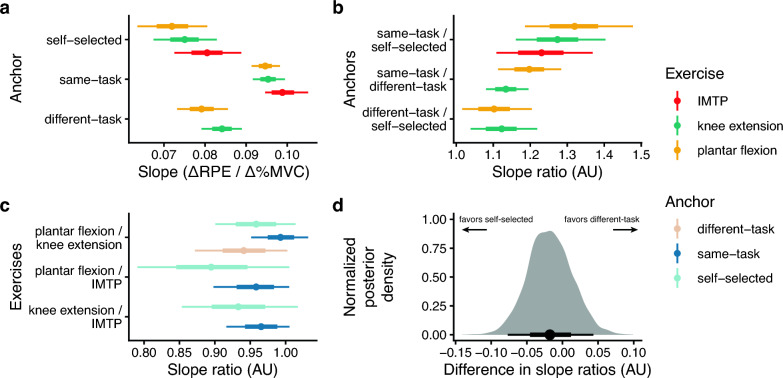
Posterior distributions of the MVC–RPE relationship for each exercise-task anchor assess the study’s predictions. Each point represents the posterior mean, and error bars indicate the 66% (thick) and 95% (thin) credible intervals. **a** The slope of each exercise–anchor pair reduces the MVC–RPE relationship into a single parameter. Slopes that are close to 0.1 indicate that RPE will be ~ 10 with 100% MVC; smaller slopes indicate that RPE will be lower for a given %MVC. The positive slopes are consistent with our first prediction, *i.e.*, that RPE will increase with relative force. **b** The slope ratios between anchors for each exercise provide insight into anchor effects. Since anchors representing harder tasks should produce lower RPEs (and thus slopes), we divided easier task slopes by harder task slopes. Indeed, consistent with the predictions, all ratios were greater than 1, indicating that anchors representing harder tasks consistently result in lower RPEs. The values of these slope ratios indicate the relative change in RPE expected from switching anchors. **c** The ratio in slopes between exercises is what we refer to as “relative exercise difficulty”. We divided the slope of the easier exercise by the slope of the harder exercise. When the same anchor was used across conditions (different-task, self-selected), we consistently observed slope ratios less than 1, indicating that the easier exercise was perceived as less effortful. **d** We compared the plantar flexion / knee extension slope ratios between the different-task and self-selected anchors. Our hypothesis implies that these should be similar. Depicted is the posterior distribution of the estimate of their difference

We used the posterior distributions of the slope estimates to assess the predictions of our study. First, RPE increased with %MVC, as demonstrated by the strictly positive slopes (Figs. 3, 4a).

Second, we evaluated the extent to which anchors representing easier tasks increased RPE scores relative to anchors representing harder tasks. To do so, we calculated the slope ratios between anchors representing easier tasks and anchors representing harder tasks (*i.e.*, $\frac{\alpha [\text{easy task}]}{\alpha [\text{hard task}]}$). Easier task anchors should result in higher RPEs and, thus, greater slopes than harder task anchors. Therefore, we would expect the calculated difference to be greater than 1. Indeed, this is what we observed: anchors representing easier tasks yielded greater slopes than those representing harder tasks, as illustrated by the slope ratios in Fig. 4b. This means that having a harder anchor, such as “the most effortful task you could imagine,” results in lower RPEs than an easier anchor, such as “the most plantar flexion effort you can exert.” The values of these slope ratios reflect the magnitude of the expected change in RPE from switching anchors. For example, in the plantar flexion task, the same-task anchor will increase RPEs by 32% (95% CrI = [19%, 48%]) relative to the self-selected anchor.

Third, we assessed how exercise demands (muscle mass) influence RPE scores. To do so, we calculated the ratio of slopes between pairs of easier tasks and harder tasks for each anchor (*i.e.*, $\frac{\alpha [\text{easy task}]}{\alpha [\text{hard task}]}$). Our prediction suggests these ratios should be less than one when evaluating slopes that share the same anchor; for example, plantar flexion would have a lower slope than IMTP when the anchor is self-selected. Indeed, when using the same anchor across exercises, exercises that required less muscle mass (numerator) generally had lower slopes than exercises requiring more muscle mass (denominator). However, this was not necessarily the case when using the same-task anchor (Fig. 4c).

Finally, to address our fourth prediction—that RPE is scale invariant and changing the anchor rescales different exercises similarly—we were constrained by the anchors that put RPE on a common scale: self-selected and imposed different-task. The latter was assessed only with unilateral plantar flexion and unilateral knee extension, so we were also constrained to perform the analysis on these two exercises. Since changing the anchor should not affect the RPE ratio between two exercises, this implies that $\frac{\alpha [\text{plantar flexion, self-selected}]}{\alpha [\text{knee extension, self-selected}]}\approx \frac{\alpha [\text{plantar flexion, different-task}]}{\alpha [\text{knee extension, different-task}]}.$ Figure 4d depicts the posterior distribution of the difference in these slope ratios. Although the posterior distribution was not centered on 0, the difference was small and fairly precisely estimated (posterior mean = − 0.02, 95% CrI = [− 0.07, 0.03]), indicating that the slope ratios across anchors were similar. In other words, changing the anchor maintained the ratio of plantar flexion to knee extension RPEs.

Notably, the findings from this section were similar after re-weighting RPE’s by force accuracies during the RPE averaging step (see Additional file 1).

### Secondary Outcomes

The complete qualitative results are presented in Supplementary Materials 3 and 4. Briefly, participants reported a range of self-selected anchors that we tentatively grouped into two main themes: (1) brief, very high-intensity lifting efforts, such as lifting a car or performing a heavy deadlift or squat, and (2) prolonged, submaximal endurance-based efforts, such as climbing a steep mountain or completing a maximal-effort run. Most participants reported having experienced their selected anchor rather than imagining it, and most used the same anchor across both the familiarization and experimental sessions. Finally, participants indicated that they were able to successfully distinguish among the different anchors and that using them influenced their ratings.

## Discussion

The present study examined how different anchoring methods (*i.e.*, same-task, different-task, and self-selected) influence RPE across three isometric exercises performed at varying intensities and involving different levels of muscle mass recruitment. Consistent with our hypothesis that RPE scores are rescaled in accordance with the anchor, we observed that (1) RPE increased with relative force level; (2) anchoring conditions affected RPE, with the same-task anchor consistently eliciting the highest RPE values, followed by the different-task anchor, and the self-selected anchor yielded the lowest ratings; (3) a consistent but trifling dose–response relationship between muscle mass and RPE ratings across shared anchors (*i.e.,* self-selected and different-task) and intensity levels; and (4) the relative differences in RPE across tasks remained similar across anchors, reflecting scale invariance.

The effects of anchors on RPE observed in the present study largely align with the findings of Malleron et al. [38], who reported that imposed anchors (same-task) yielded higher RPE values than self-selected anchors. However, the magnitude of this effect was smaller in the present study. Several factors may contribute to this discrepancy, including variations in exercise type and intensity. Notably, the differences in RPE values between anchor types became more pronounced at higher intensities (80% and 100% of MVC), which is consistent with Malleron et al.’s exclusive use of maximal effort trials. Beyond partially replicating Malleron et al.’s results, our study offers two key methodological contributions. First, while Malleron et al. focused solely on maximal contractions, our protocol spanned a broader range of intensities (20–100% MVC). This enabled finer differentiation across anchor types and demonstrated their effects beyond a single intensity level. Second, we introduced a novel different-task anchor, wherein participants rated their effort based on a task (IMTP) different from the one being performed. This anchor consistently produced RPE values that fell between those of the same-task and self-selected anchors.

The fact that participants reported different RPE scores depending on the anchor, despite producing nearly identical forces, suggests that RPE is not a straightforward reflection of the exerted objective effort. Rather, the ratings appear to reflect a comparison process in which the experienced intensity is scaled relative to the session-specific anchor. This interpretation is consistent with comparison-based theories of judgment, which posit that individuals rarely assess stimuli in isolation or by referencing objective standards [45–50]. Instead, evaluations are made relative to internal reference points, which are informed by memory, context, and psychoeconomic cues [45–50]. This comparison process is particularly sensitive to recent, salient, or personally meaningful experiences [47–49]. In this context, the top anchor on the RPE scale functions as a reference value, framing all subsequent evaluations. Different anchoring conditions (same-task, different-task, or self-selected) likely triggered distinct internal comparisons, thereby systematically influencing or rescaling the rating of perceived effort reported in our study.

We observed a smaller-than-expected dose–response relationship between muscle mass involvement and RPE ratings across all anchoring conditions. We speculate that the following three reasons might partly explain this result: (1) the tasks chosen, (2) the high cognitive load associated with the experimental protocol, and/or (3) the experimental context. First, Malleron et al. studied two extreme tasks: an isometric squat and a hand gripper task. The difference in objective effort between these two tasks may have been greater than that between the three tasks performed in this study, resulting in greater RPE discrepancies in Malleron et al. Second, participants in this study were required to comprehend complex anchoring procedures, recall the anchor’s meaning on each trial, apply and monitor force outputs in real-time, and report their RPE repeatedly, all while keeping the differences between exercises in mind. This cognitively demanding combination may have resulted in some form of overload, leading participants to focus primarily on interpreting the anchor within each exercise, thereby somewhat negating the differences between exercises. Hence, our design might have potentially obscured differences that might have emerged under simpler protocols. Finally, whereas participants in Malleron et al.’s study performed repeated maximal-effort repetitions without visual feedback, those in the current study completed repeated submaximal-effort repetitions with visual feedback. This difference in task demands may have led to distinct calibrations and, consequently, dissimilar ratings. Specifically, participants’ ratings in both studies could have been shaped by their ongoing experience within the protocol (*e.g.,* familiarization, earlier sets/reps, visual feedback) and their expectations for subsequent trials. Taken together, this suggests that the experimental context should be viewed as a source of bias and a potential driver of heterogeneity across studies.

The secondary qualitative data nicely complemented our quantitative results. First, we documented which anchors participants adopted to represent maximal effort in the self-selected condition and identified two primary themes: (1) lifting or moving a heavy object and (2) sustaining a prolonged endurance effort. Yet, even within these themes, anchors differed across participants, demonstrating between-individual heterogeneity that may influence perceived-effort ratings and their comparability. Second, most participants reported retaining the same anchor across sessions, indicating within-individual stability, and most referenced previously experienced tasks rather than imagined ones (see Additional file 1). Third, participants reported that they could clearly distinguish between the three anchors and that using them influenced their ratings, primarily by contextualizing and calibrating their perceived effort (see Additional file 1). Collectively, these descriptive data characterize the anchors people naturally select and clarify how open-ended Borg-style instructions are interpreted in practice.

Several methodological considerations should be acknowledged when interpreting our results. First, our RPE approach differed from Borg's, which is the most commonly used approach in exercise science. We employed a different definition of perceived effort [17] and used a modified visual numerical RPE scale (without verbal descriptors), consistent with prior research in our laboratory [3, 38, 51]. While this may have improved discriminability and reduced interpretive bias [1], it may preclude valid comparisons with RPE studies that do not incorporate these elements [1, 20]. Second, while the isometric contractions enabled consistent and comparable force application, their ecological validity is uncertain. Future studies should assess whether anchoring effects generalize to dynamic, real-world tasks. Finally, the sample included only resistance-trained participants.

### Practical Applications

These findings have direct implications for the everyday use of RPE in resistance training. Practitioners should standardize anchoring across sessions when using RPE for prescription or monitoring. For example, coaches can establish a shared reference task and a clear definition of “10” early in a training block, then repeat the same instruction periodically and whenever the training context changes. This is especially relevant when tracking trends over time or comparing RPE across exercises, because shifts in anchoring can alter ratings that reflect reframing rather than changes in training load or subjective difficulty. Our slope-ratio approach may be a valuable tool for facilitating comparisons of tasks or exercises. By comparing the slopes from two exercises sharing the same anchor, we estimated what may be interpreted as a metric for the “relative difficulty” of the exercises. For example, a slope ratio of 0.9 would indicate that the exercise in the numerator is 10% “easier” than the exercise in the denominator. Explicitly placing RPEs from different exercises on the same scale may enable practitioners to select exercises based on their relative difficulty. This exciting direction remains an avenue for future inquiry.

## Conclusion

We demonstrated that altering the upper anchor has a meaningful and rational effect on RPE values across intensities, with a minor and uncertain dose–response relationship between RPE and muscle mass. These results underscore the importance of clearly defining and consistently reporting anchoring procedures. Researchers and practitioners should align anchor choices with their goals and report them transparently to support valid comparisons and interpretations.

## Supplementary Information

Additional file 1.

## Data Availability

The datasets used in the current study, together with the R statistical analysis code, are available online at: https://osf.io/jsehg/files/osfstorage.

## Abbreviations

RPE: Rating of perceived effort
MVC: Maximal voluntary contraction
IMTP: Isometric mid-thigh pull
RT: Resistance-training
PF: Isometric plantar flexion
KE: Isometric knee extension
