# Supplementary material for: Modifying the Meaning of the Upper Anchor Predictably Shapes Ratings of Perceived Effort: A Randomized Crossover Trial
Source: Sports Med Open. 2026 Jun 6;12:63. doi: 10.1186/s40798-026-01040-x (PMC13242339; doi:10.1186/s40798-026-01040-x)
Supplement: Supplementary file 1 — Additional file 1. [file 40798_2026_1040_MOESM1_ESM.docx]

**Supplemental materials**

### **Supplemental material 1**

- **Expanding on the three tools we used to measure force production in the study**
  *Isometric plantar flexion (PF) and isometric knee extension (KE).* PF and KE were performed using an isokinetic dynamometer (Computer Sports Medicine, Inc., Stoughton, MA, USA), with participants lying prone (PF) or seated (KE). Force production for both PF and KE were recorded at a frequency of 500 Hz using the HUMAC NORM Isokinetic software (Computer Sports Medicine, Inc., Stoughton, MA, USA). The raw torque (N·m) signals were processed using the system’s internal digital filtering algorithms to minimize mechanical noise and ensure signal stability prior to analysis. The HUMAC NORM stands as the gold standard for the measurement of force production in an isokinetic setting [1]. Both the target absolute force (with a ±5% tolerance range from the target %MVC) and the participant’s ongoing absolute force output were displayed as horizontal lines on a screen to aid participants in achieving the prescribed force levels. Accordingly, when viewing the *y*-axis displaying absolute force for each contraction, it would have been difficult to infer the corresponding percentage of MVC, thereby preventing them from aligning their RPEs with expected values (*e.g.,* associating 60% of MVC with an RPE of 6). To standardize and increase measurement accuracy, participants were securely positioned with the dominant leg (active limb) attached to the dynamometer using appropriate adapters, as specified by the exercise and according to the manufacturer's instructions. The active limb was immobilized to minimize extraneous movement. To ensure consistent force application, before each contraction, slack was removed, and participants were instructed to apply force steadily, against the plate (PF) or pad (KE), without explosive movements. The chair's height, reclining angle, and distance from the dynamometer, as well as the dynamometer’s rotation angle, height, and adapter positioning, were individually adjusted to align with the participant’s anatomical axes. These settings were recorded during the familiarization session and replicated throughout the experimental sessions.

### *Isometric mid-thigh pull (IMTP)***.** IMTP was performed with participants standing on a portable force plate equipped with a fixed handle (Deltas, Kinvent, Orsay, France), recording ground reaction force (N) at 500 Hz via the K-Force software (version 5.4.9, Kinvent, Orsay, France). The tool was used in prior research in our laboratory and was found to be reliable and valid for measuring force output in IMTP [2]. The absolute force output was displayed on a circular scale with numerical values, and before each contraction participants were verbally informed of the required target force. This setup ensured real-time visual feedback and accurate monitoring throughout the protocol. To ensure consistent force application, participants were instructed to eliminate any slack from the handle before each contraction, and then exert force by pulling on the handle while simultaneously pushing into the ground. To standardize and increase measurement accuracy, The handle height was adjusted to align with the midpoint of the participant’s thigh, as measured during familiarization. This height was documented and replicated across all experimental sessions.


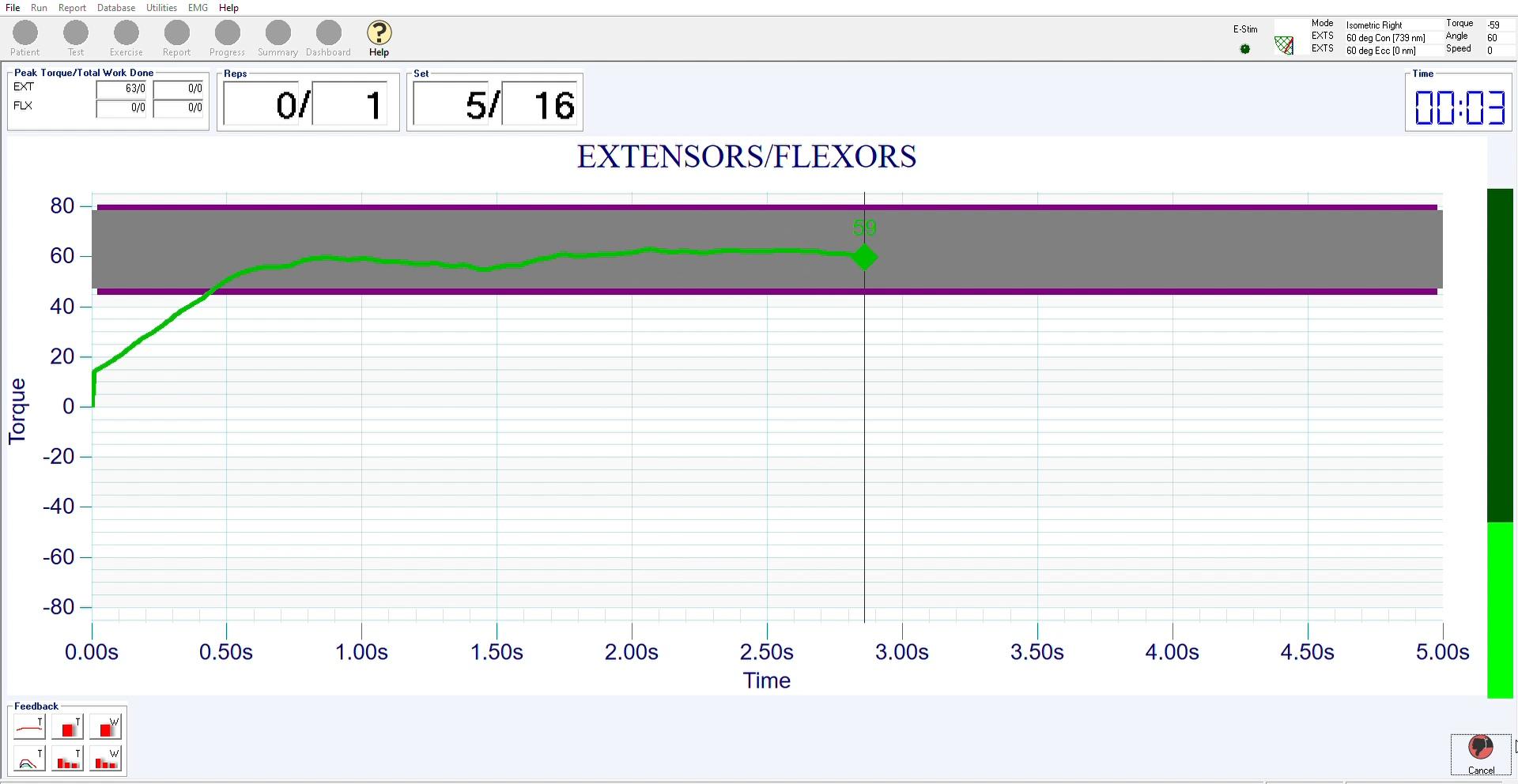


HUMAC NORM isokinetic dynamometer screen while performing an isometric plantar flexion or isometric knee extension repetition/contraction ([demonstration](https://www.youtube.com/watch?v=iEfR4q3_cUM)).


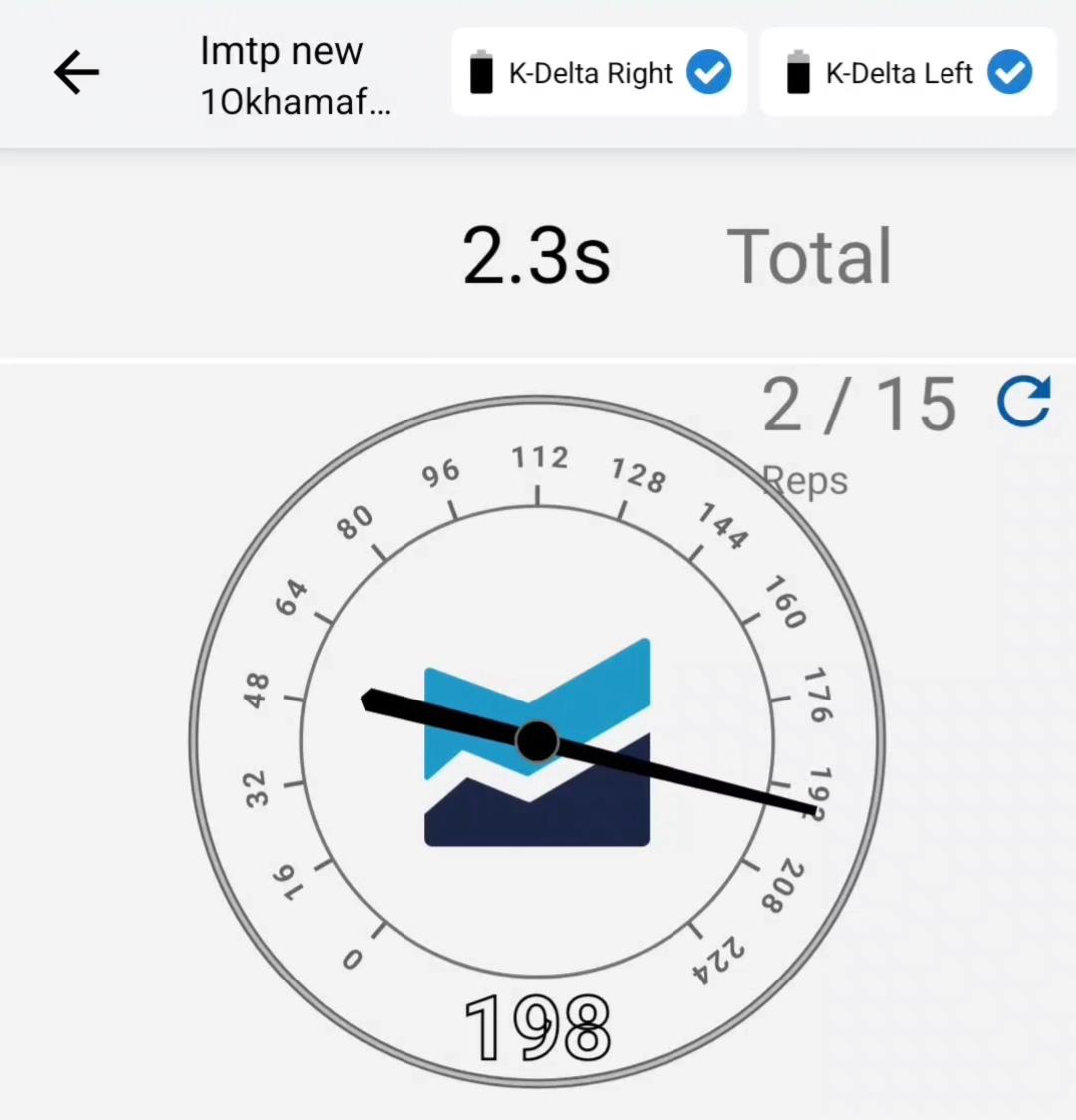


K-Delta force plate (Kinvent, Orsay, France) screen while performing an IMTP repetition/contraction ([demonstration](https://www.youtube.com/shorts/Sr75LgPQb2w)).

1. Whinton AK, Thompson KMA, Power GA, Burr JF. Testing a novel isokinetic dynamometer constructed using a 1080 Quantum. PLoS One. 2018;13(8):e0201179.

2. Boxman-Zeevi Y, Schwartz H, Har-Nir I, Bordo N, Halperin I. Prescribing Intensity in Resistance Training Using Rating of Perceived Effort: A Randomized Controlled Trial. Front Physiol. 2022;13:891385.

**Supplemental material 2**

**Definitions and Explanations of effort, perception of effort, RPE scale, and the three anchors used in the study**

During the familiarization session, participants received a 25-minute instructional presentation based on the recommendations of Halperin and Vigotsky [1] regarding the introduction and use of RPE scales.

We began with abstract definitions: *effort* was defined as “the energy utilized to perform an action,” and *perception of effort* as “the instantaneous experience of utilizing energy to perform an action.” Participants were then introduced to a vertically ascending 0–10 RPE scale translated into Hebrew, where 0/10 indicated no effort (e.g., “sitting in a chair”) and 10/10 indicated maximal effort (e.g., “exerting force to the point of momentary failure”). The scale contained only consecutive numerical values, without accompanying verbal descriptors (e.g., "hard"), and participants were allowed to report fractions as their ratings. This version of the scale has been used in previous studies conducted in our lab [2–4]. We chose this format for several reasons. First, because participants needed to keep their visual attention on the force feedback display, the RPE scale was not continuously visible. We therefore assumed that recalling specific verbal labels tied to particular numbers might be more difficult immediately after each repetition. In contrast, relying primarily on a single upper anchor as the reference point likely reduces memory demands and cognitive load. Second, we speculate that this format reduces the tendency to cluster responses around particular verbal anchors, a pattern often observed with scales that include intermittent descriptors [5–8]. Finally, strong associations among commonly used RPE scales [5,6,9–11] suggest that different scales generally yield comparable ratings.

## **Rating of perceived effort (RPE) scale - *English* version and *Hebrew* version**

***
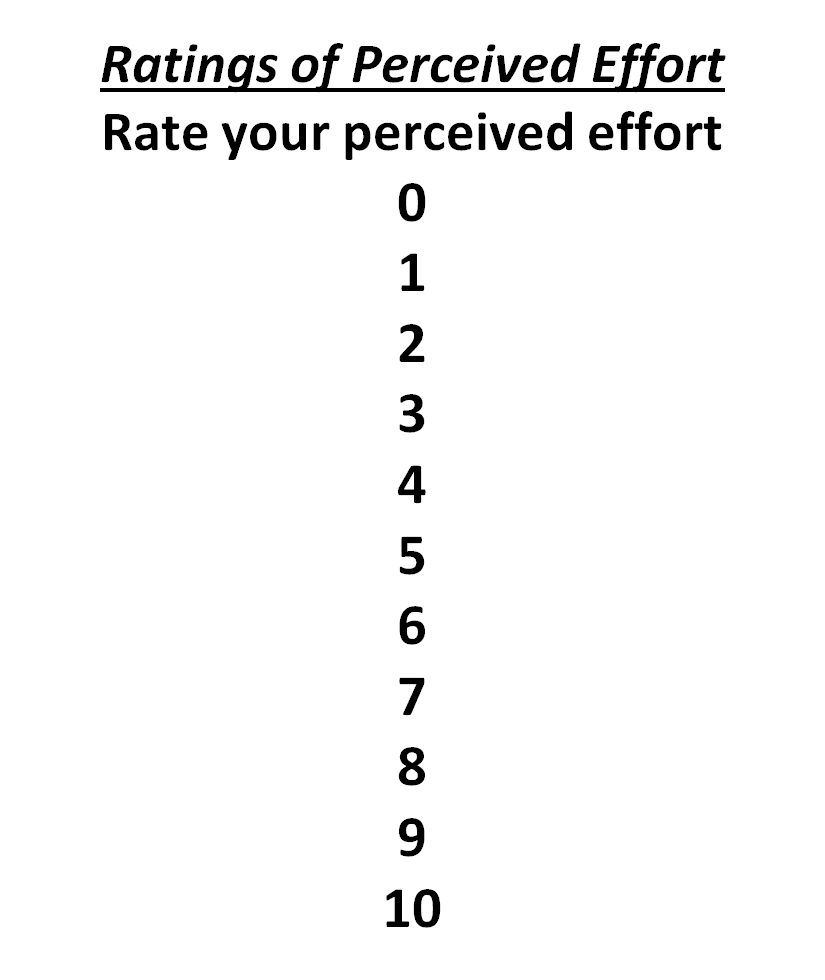

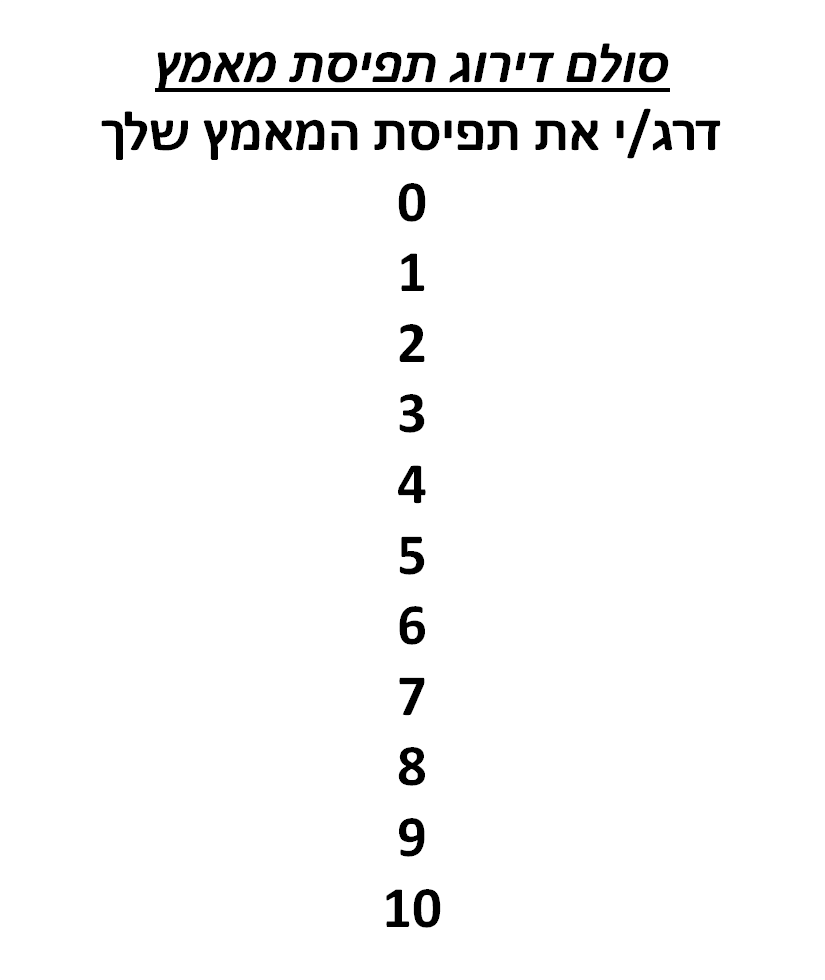
***

Participants were then taught that the 10/10 effort may depend on the anchoring condition. To illustrate, they were asked to imagine performing a dumbbell curl to task failure and to rate the effort of their most recent repetition:

- **Same-task anchor.** Here, 10/10 was defined as the maximal effort required to reach momentary failure in the same exercise being performed. For the curl example, participants rated their perceived effort relative to the effort required to fail in the curl itself.
- **Different-task anchor.** In this case, 10/10 was defined as the maximal effort exerted in a different exercise, specifically, an isometric mid-thigh pull (IMTP) performed at maximal intensity. Participants rated the curl repetition relative to this IMTP-based reference effort.
- **Self-selected anchor.** Here, 10/10 corresponded to the most effortful task the participant had ever experienced or could imagine performing. Ratings were anchored to this personal or imagined “greatest effort” reference.

Finally, the explanation was contextualized to the study exercises. Participants were instructed to mentally replace the dumbbell curl example with exerting maximal force in the plantar flexion (PF), knee extension (KE) and the IMTP tasks. They were then guided to report RPE anchored to one of the three defined anchors. To ensure understanding, these explanations were revisited and reinforced during both the familiarization and experimental sessions.

**References**

1. Halperin I, Vigotsky AD. An Integrated Perspective of Effort and Perception of Effort. Sports Med Auckl NZ. 2024;54:2019–32. https://doi.org/10.1007/s40279-024-02055-8

2. Ben-Ari A, Silverman Y, Obolski U, Halperin I. On Your Mark, Get Set, Choose! A Randomized Cross-Over Study Comparing Fixed and Self-Selected Rest Periods in Interval Running Among Professional Female Soccer Players. Sports Med - Open. 2025;11:2. https://doi.org/10.1186/s40798-024-00803-8

3. Kobi O, Ben-Ari A, Dello Iacono A, Silverman Y, Obolski U, Halperin I. Prescribing playing intensity in small-sided games using rating of perceived effort among youth soccer players: a randomized crossover trial. Sci Med Footb. 2025;1–7. https://doi.org/10.1080/24733938.2025.2471318

4. Malleron T, Har-Nir I, Vigotsky AD, Halperin I. Rating of perceived effort but relative to what? A comparison between imposed and self-selected anchors. Psychol Sport Exerc. 2023;66:102396. https://doi.org/10.1016/j.psychsport.2023.102396

5. Borg E, Kaijser L. A comparison between three rating scales for perceived exertion and two different work tests. Scand J Med Sci Sports. 2006;16:57–69. https://doi.org/10.1111/j.1600-0838.2005.00448.x

6. Fanchini M, Ferraresi I, Modena R, Schena F, Coutts AJ, Impellizzeri FM. Use of CR100 Scale for Session Rating of Perceived Exertion in Soccer and Its Interchangeability With the CR10. Int J Sports Physiol Perform. 2016;11:388–92. https://doi.org/10.1123/ijspp.2015-0273

7. Johnson MJ, Close L, Gillon SC, Molassiotis A, Lee PH, Farquhar MC, et al. Use of the modified Borg scale and numerical rating scale to measure chronic breathlessness: a pooled data analysis. Eur Respir J. 2016;47:1861–4. https://doi.org/10.1183/13993003.02089-2015

8. Aucoin R, Ekström M, Li P, Bourbeau J, Jensen D. A response bias exists away from Borg Category-Ratio 0-10 scale values without associated verbal descriptors for symptom intensity ratings during cardiopulmonary exercise testing. 2024. p. PA2604. https://doi.org/10.1183/13993003.congress-2024.PA2604

9. Grant S, Aitchison T, Henderson E, Christie J, Zare S, McMurray J, et al. A comparison of the reproducibility and the sensitivity to change of visual analogue scales, Borg scales, and Likert scales in normal subjects during submaximal exercise. Chest. 1999;116:1208–17. https://doi.org/10.1378/chest.116.5.1208

10. Robertson RJ, Goss FL, Dube J, Rutkowski J, Dupain M, Brennan C, et al. Validation of the adult OMNI scale of perceived exertion for cycle ergometer exercise. Med Sci Sports Exerc. 2004;36:102–8. https://doi.org/10.1249/01.MSS.0000106169.35222.8B

11. Arney B, Glover R, Fusco A, Cortis C, de Koning J, Erp T, et al. Comparison of rating of perceived exertion scales during incremental and interval exercise. Kinesiology. 2019;51:150–7. https://doi.org/10.26582/k.51.2.1

**Supplemental material 3**

**The tasks participants selected as their anchors under the self-selected anchor session - Sorted by theme**

| **Gender** | **Themes** | **Which task did you recall or imagine to represent maximal effort?** | **Was the task experienced or imagined?** | **In familiarization, did you use the same anchor?  If not, which task was it?** |
| --- | --- | --- | --- | --- |
| M | Lifting  activities | Trying to lift the car off the floor | Imagined | Same task |
| M |  | Lifting a heavy weight off the floor | Experienced | Same task |
| M |  | Back squat with about 250+ kg | Imagined | Same task |
| M |  | Leg press with a heavy load while already exhausted | Experienced | Same task |
| M |  | Lifting my grandfather (piggyback lift) | Experienced | Same task |
| M |  | Bench pressing maximum load | Experienced | Same task |
| M |  | A 110 kg bench press | Experienced | Switched from climbing a mountain to bench press. |
| F |  | Hex bar deadlift with maximum load | Experienced | Same task |
| F |  | A 100 kg deadlift | Experienced | Same task |
| F |  | Lifting a boulder out of the ground | Imagined | Same task |
| F |  | Carrying a washing machine | Experienced | Switched from carrying a cabinet  to carrying a washing machine |
| F |  | Heavy deadlift | Imagined | Same task |
| F |  | A 90-100 kg squat and a 105 kg deadlift | Deadlift - imagined  Squat - experienced | Same task |
| F |  | Pull-up with a weight on my chest | Imagined | Same task |
|  | | | | |
| M | Aerobic activities | Walking at a high pace for 48 hours with an added 110% of body weight. | Experienced | Same task |
| M |  | 5 km run with the goal of breaking a personal record | Experienced | Same task |
| M |  | A HIIT session composed of 21 aerobic and strength exercises using a 20:5-second work-to-rest ratio | Experienced | Same task |
| M |  | Climbing a steep mountain with an added 45 kg after a 12-kilometer walk in extreme terrain with the load | Experienced | Same task |
| M |  | A HIIT session with several exercises | Experienced | Same task |
| M |  | A trek in Nepal to the Everest Base Camp, focused on the ascent | Experienced | Same task |
| F |  | A maximal effort 4-kilometer run | Experienced | Switch from a heavy clean exercise to a run. |
| F |  | Intense jumping rope session | Experienced | Same task |
| F |  | A Running race where I took 12th place and went all out | Experienced | Same task |
| F | Other activities | Delivering my 3rd baby without an epidural | Experienced | Same task |
| F |  | Performing cardiopulmonary resuscitation | Experienced | Same task |
| F |  | Hopping up the stairs on one leg after a leg injury | Experienced | Same task |

* It should be noted that the main points are presented here, and not the text in its entirety as written by the participants, so as not to overload the reading. For more information, please see the shared research data file (xlsx).

**Supplemental material 4**

**Final session Questionnaire - Sorted by gender**

| **on a 7-point Likert scale (0 = not at all; 6 = very much) to what extent you could distinguish between:** | Range (Median) |
| --- | --- |
| Same and different task anchors? | 3-6 (5) |
| Same task and self-selected anchors? | 3-6 (5) |
| Different task and self-selected anchors? | 3-6 (5) |
|  | |
| **To what extent do you think using a certain anchor (same task, different task of self-selected) affected your perception of effort in real-time when performing the exercises?** | |
| **Female (n=13)** | |
| - The same task anchor influenced me the most. It helped me aim for maximal effort and better evaluate my perception of effort. The self-selected anchor, since it resembled the deadlift, specifically helped me gauge effort during that IMTP. | |
| - Yes, the comparison between the same task and the self-selected/different task affected my ratings. | |
| - The same task anchor was closest to capturing the effort at that moment. The self-selected anchor was more difficult because I relied on memory and feelings I had felt in the past. | |
| - The same task anchor had the strongest effect on my perception of effort. It was the hardest to compare my effort to the self-selected anchor, and the easiest to compare it to the same-exercise anchor, which felt the most precise. The different-task anchor partially affected my perception but required more concentration. | |
| - I felt the anchors influenced my perception mainly when effort levels were high. It was easier to rate according to the requested anchor in those cases. Each time before rating, I tried to recall my maximal effort for the given anchor, so the chosen anchor definitely influenced my perception of effort. With the same-task, it was easier to gauge the relation between maximal and current effort across the 16 repetitions, whereas with the other anchors, it was more complex. | |
| - Had a slight influence. | |
| - It had a strong impact, as it required assessing the effort relative to each anchor while ignoring other sensations, such as discomfort, and focusing only on the exertion itself. | |
| - Using a specific anchor in real-time strongly affected my perception of effort and helped me distinguish between levels of exertion and how I experienced them relative to one another. | |
| - The self-selected anchor felt less concrete compared to the same or different task anchors. It was the hardest one to estimate. | |
| - Had a moderate influence. | |
| - In the same task anchor, it was easier to compare the effort. | |
| - Helped a lot. | |
| - I believe it had a strong impact. | |
| **Male (n=13)** | |
| - I think it helped. It made my ratings more accurate than if I had no anchor at all. | |
| - Using a specific anchor had a strong positive effect on performance and provided a personal benchmark for my ability. | |
| - It slightly affected me in the same and different task anchors; the self-selected anchor had more real-time influence on my perception of effort. | |
| - It influenced my perception because the anchor varied. | |
| - In my opinion, it had a strong impact. I also tried something extra to differentiate between them: with the self-selected anchor, I compared the maximal reps I did at the start of each exercise to those in the self-selected anchor; with the different-exercise anchor, I did the same. This helped clarify differences. Of course, it’s hard to separate factors like fatigue or soreness, but I did my best to try. | |
| - I think the self-selected anchor had the strongest influence, followed by the different and same task anchors (the last two affected me differently but similarly in magnitude). Overall, the differences weren’t huge in absolute effort perception, but they were definitely noticeable. | |
| - The different and self-selected anchors slightly delayed my response since I had to compare them in my head first. Aside from that minor delay, it didn’t significantly affect their perception. | |
| - Using the anchors helped me understand the effort I put into each exercise. | |
| - No effect. After performing, I rated based on my general feeling relative to each anchor. | |
| - Strong influence. It clarified how much effort I was exerting in relation to my maximal effort. | |
| - On a scale of 1–10, about 7. It was easiest to assess effort with the same task anchor. | |
| - Strong influence. | |
| - Strong influence. | |

**Supplemental material 5**

**Model 1**


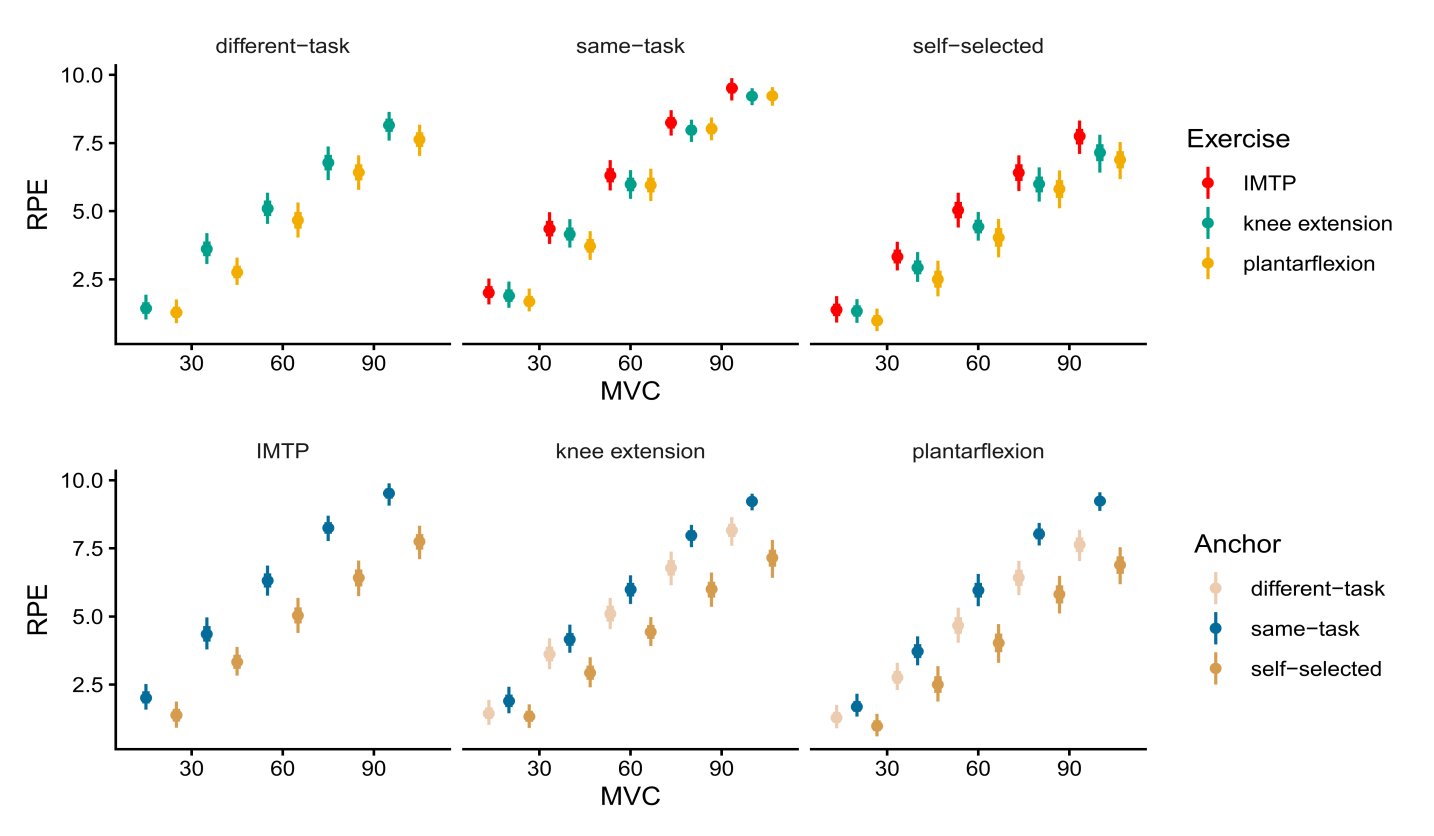


**Posterior distributions illustrate RPE’s dependence on %MVC, anchor, and task.** Each point represents the posterior median, and error bars indicate the 66% (thick) and 95% (thin) credible intervals. *Nota bene* many of the 66% credible intervals are very close to the point estimates and, therefore, are difficult to see. (**Top row**) Conditioning each facet on anchor emphasizes the effect of exercise (color), (**bottom row**) while conditioning each facet on exercise emphasizes the effect of anchor (color). Generally, the effects of exercise appeared modest (different-task and self-selected) to negligible (same-task), while the anchor effects were more dramatic.

**Supplemental material 6**

**Accuracy-weighted sensitivity analyses**

**
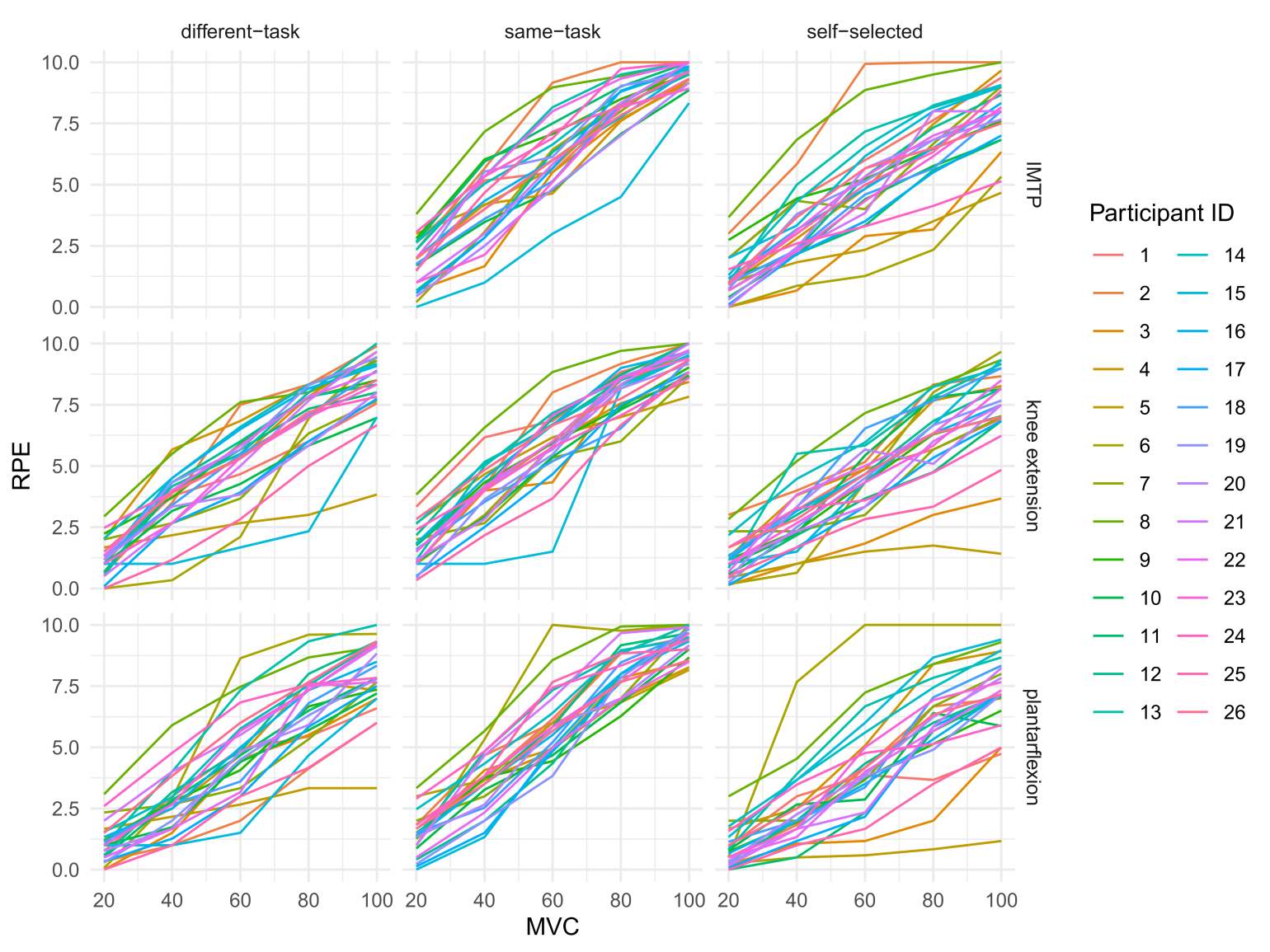
**

**Depictions of individual-level RPE–MVC relationships.** We derived accuracy-weighted means for each subject over each exercise-anchor-intensity triple. Specifically, $RPE=\frac{\sum a_{i}\mathrm{RPE}_{i}}{\sum a_{i}}$, where the accuracy $a_{i}$ of repetition $i$ is the inverse of the mean squared error of the force trace (deviation from the target force) between 1 sec and 4 sec, *i.e.*,

$$a_{i}=\left( T^{-1}\sum_{t=1s}^{4s} \left( \mathrm{force}\left( t \right)-target \right)^{2} \right)^{-1}.$$

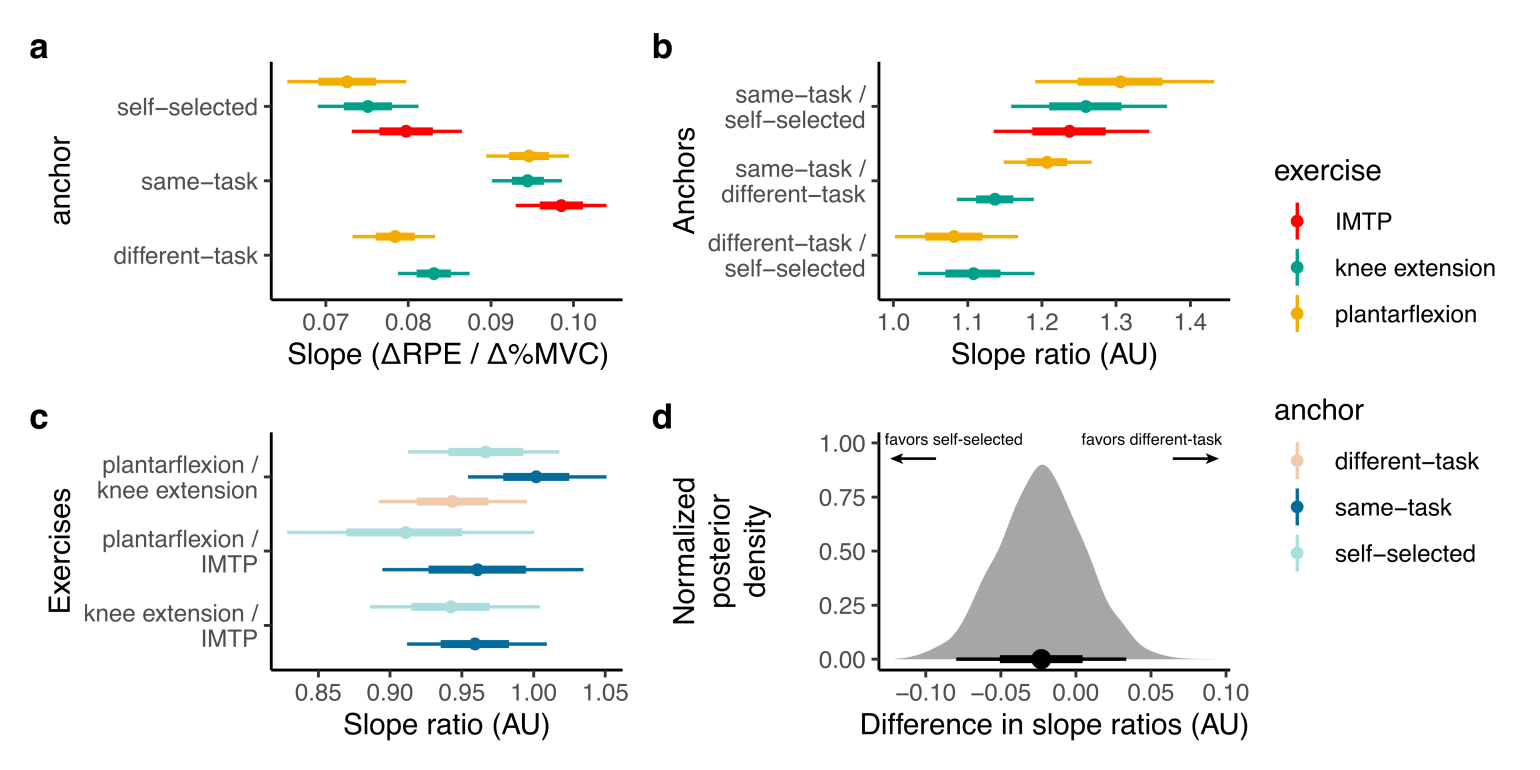


**Posterior distributions of the accuracy-weighted MVC–RPE relationship for each exercise-task anchor assess the study’s predictions.** Each point represents the posterior mean, and error bars indicate the 66% (thick) and 95% (thin) credible intervals. (a) The slope of each exercise-anchor pair reduces the MVC–RPE relationship into a single parameter. Slopes that are close to 0.1 indicate that RPE will be ~10 with 100% MVC; smaller slopes indicate that RPE will be lower for a given %MVC. The positive slopes are consistent with our first prediction, *i.e.*, that RPE will increase with relative force. (b) The slope ratios between anchors for each exercise provide insight into anchor effects. Since anchors representing harder tasks should produce lower RPEs (and thus slopes), we divided easier task slopes by harder task slopes. Indeed, consistent with the predictions, all ratios were greater than 1, indicating that anchors representing harder tasks consistently result in lower RPEs. The values of these slope ratios indicate the relative change in RPE expected from switching anchors. (c) The ratio in slopes between exercises is what we refer to as “relative exercise difficulty.” We divided the slope of the easier exercise by the slope of the harder exercise. When the same anchor was used across conditions (different-task, self-selected), we consistently observed slope ratios less than 1, indicating that the easier exercise was perceived as less effortful. (d) We compared the plantar flexion / knee extension slope ratios between the different-task and self-selected anchors. Our hypothesis implies that these should be similar. Depicted is the posterior distribution of the estimate of their difference.
